# Silver Ion High-Performance Liquid Chromatography—Atmospheric Pressure Chemical Ionization Mass Spectrometry: A Tool for Analyzing Cuticular Hydrocarbons

**DOI:** 10.3390/molecules28093794

**Published:** 2023-04-28

**Authors:** Vladimír Vrkoslav, Petra Horká, Jindřich Jindřich, Miloš Buděšínský, Josef Cvačka

**Affiliations:** 1 Institute of Organic Chemistry and Biochemistry of the Czech Academy of Sciences, Flemingovo Náměstí 542/2, 160 00 Prague, Czech Republic; vladimir.vrkoslav@uochb.cas.cz (V.V.); peta.machajda@gmail.com (P.H.); milos.budesinsky@uochb.cas.cz (M.B.); 2 Department of Analytical Chemistry, Faculty of Science, Charles University, Hlavova 2030/8, 128 00 Prague, Czech Republic; 3 Department of Organic Chemistry, Faculty of Science, Charles University, Hlavova 2030/8, 128 00 Prague, Czech Republic; jindrich.jindrich@natur.cuni.cz

**Keywords:** double bonds, hydrocarbons, mass spectrometry, *Neobellieria bullata*, *Periplaneta americana*, semiochemicals

## Abstract

Aliphatic hydrocarbons (HCs) are usually analyzed by gas chromatography (GC) or matrix-assisted laser desorption/ionization (MALDI) mass spectrometry. However, analyzing long-chain HCs by GC is difficult because of their low volatility and the risk of decomposition at high temperatures. MALDI cannot distinguish between isomeric HCs. An alternative approach based on silver ion high-performance liquid chromatography (Ag-HPLC) is shown here. The separation of HC standards and cuticular HCs was accomplished using two ChromSpher Lipids columns connected in series. A gradient elution of the analytes was optimized using mobile phases prepared from hexane (or isooctane) and acetonitrile, 2-propanol, or toluene. HCs were detected by atmospheric pressure chemical ionization mass spectrometry (APCI-MS). Good separation of the analytes according to the number of double bonds, cis/trans geometry, and position of double bonds was achieved. The retention times increased with the number of double bonds, and trans isomers eluted ahead of cis isomers. The mobile phase significantly affected the mass spectra of HCs. Depending on the mobile phase composition, deprotonated molecules, molecular ions, protonated molecules, and various solvent-related adducts of HCs were observed. The optimized Ag-HPLC/APCI-MS was applied for characterizing cuticular HCs from a flesh fly, *Neobellieria bullata*, and cockroach, *Periplaneta americana*. The method made it possible to detect a significantly higher number of HCs than previously reported for GC or MALDI-MS. Unsaturated HCs were frequently detected as isomers differing by double-bond position(s). Minor HCs with trans double bonds were found beside the prevailing cis isomers. Ag-HPLC/APCI-MS has great potential to become a new tool in chemical ecology for studying cuticular HCs.

## 1. Introduction

Aliphatic hydrocarbons (HCs) are the main constituents of petroleum. They are also biosynthesized by eukaryotic [1,2,3,4] and prokaryotic [5] organisms. Aliphatic HCs are commonly found in the waxy cuticular layer on the surface of higher plants and insects. HCs in plant epicuticular waxes help to decrease surface wetting or water loss and protect plants from ultraviolet light [1]. Insect cuticular HCs are usually complex mixtures of n-alkanes, mono-, di-, trimethyl alkanes, and unsaturated HCs with cis double bonds. Besides their main function in cuticle protection, the genetically encoded mixtures of cuticular HCs often serve as semiochemicals [2,3,4].

HCs are usually analyzed by gas chromatography (GC), an excellent and time-proven method for volatile and semivolatile compounds. However, GC has fundamental limitations for long-chain and highly unsaturated HCs. High-temperature columns can overcome some limitations [6,7]; however, problems related to decomposition in the injectors or limited volatility causing excessive peak broadening at high retention times cannot be avoided completely. The ability of electron ionization (EI) mass spectrometry to identify HCs is limited because heavier species provide weak or no molecular ions [8]. The intensities of molecular ions are greatly enhanced in EI with supersonic molecular beams [9,10], but this technique is not widely available. Chemical ionization MS can also be used, but it is less sensitive and incompatible with library-based sample identification.

Besides GC and GC/MS, several other methods have been applied to analyze long-chain hydrocarbons, including field desorption and ionization [11,12,13,14], negative ion electrospray ionization (after derivatization) [15], or laser-induced acoustic desorption combined with CI [16,17]. Matrix-assisted laser desorption/ionization mass spectrometry (MALDI-MS) was used for the analysis of synthetic polymers and HCs [18,19,20,21,22], and this technique made it possible to discover long-chain HCs in insects [23]. Silver-assisted laser desorption/ionization mass spectrometry (Ag-LDI-MS) was used for very long-chain cuticular HCs characterization [24]. Direct analysis in real time (DART) MS was applied for the analysis of HCs in both positive [25] and negative [26] ion modes. Helium ionization MS, a technique similar to DART, was used to analyze aliphatic HCs [27]. Unfortunately, these methods are difficult to interface with liquid-based chromatographic separations.

A chromatographic analysis of HCs using HPLC is advantageous because the separation occurs at significantly lower temperatures than in GC. Hence, thermally less stable hydrocarbons, including those with multiple double bonds and very long chains, can also be analyzed. Optimizing the mobile phase’s composition makes separating HCs, even those of high molecular weight, possible. Surprisingly few papers have focused on the HPLC of aliphatic HCs so far. The published papers mostly deal with isolating HC fractions using normal phase, silver ion chromatography (Ag-HPLC), or exceptionally reversed-phase HPLC [28,29,30,31,32]. Ag-HPLC is an established method used mainly in lipid analysis [33,34]. The separation is based on the interactions of double-bond electrons with silver ions and polar interactions with column support material. Ag-HPLC separates lipids according to the number, position, and geometry of double bonds, and the strength of the interaction (retention) increases with the number of double bonds [34,35]. The technique was previously used to separate neutral lipids such as fatty acid esters [36,37,38,39,40] and triacylglycerols [41,42,43,44]. As regards hydrocarbons, retention times of the synthetic n-alkadiene standards were studied in an Ag-HPLC system [32].

The main challenge for analyzing hydrocarbons by HPLC is detection. Most common HPLC detectors are insensitive to aliphatic HCs lacking strong chromophores or ionizable functional groups. HCs have been detected by universal evaporative light-scattering detectors [45], indirect photometric detection [30], refractometric detection [32], or dielectric constant detection [29]; however, none of these detectors provide structural information. Mass-spectrometric detection of HCs can be achieved using atmospheric pressure chemical ionization (APCI). Pentane, hexane, heptane, cyclohexane, isooctane [14,46,47,48,49,50,51], carbon disulfide [51,52] solvents, and reagent gases were shown to ionize saturated HCs. Besides commonly formed [M − H]^+^ ions, multiplying the branched and unsaturated HCs produced [M − 2H]^+·^ and [M + H]^+^, respectively [46]. Toluene enhanced the formation of positively charged molecular ions in APCI [53]. To our knowledge, only one report in the literature deals with the APCI detection of HCs in HPLC [46].

In this work, an Ag-HPLC/APCI-MS method for analyzing long-chain HCs was developed and applied for analyzing insect cuticular HCs. The results are compared to previously published data from GC/MS and MALDI-MS experiments. The advantages and limitations of the new approach are discussed later in the paper.

## 2. Results and Discussion

### 2.1. Ag-HPLC of Hydrocarbons

The HPLC conditions were optimized to achieve good separation of HCs. Since long-chain unsaturated hydrocarbons were not commercially available, we used a mixture of cuticular HCs isolated from the flesh fly *N. bullata*. The composition of the fly cuticular HCs is known; the mixture contained aliphatic HCs with up to 46 carbons and 0–3 double bonds [7,23,54,55]. Insects produce unsaturated HCs mainly with cis double bonds, which would not allow us to optimize the method fully. Therefore, we also used a synthetic mixture of six monounsaturated HCs with cis and trans double bonds.

**Figure 1:**
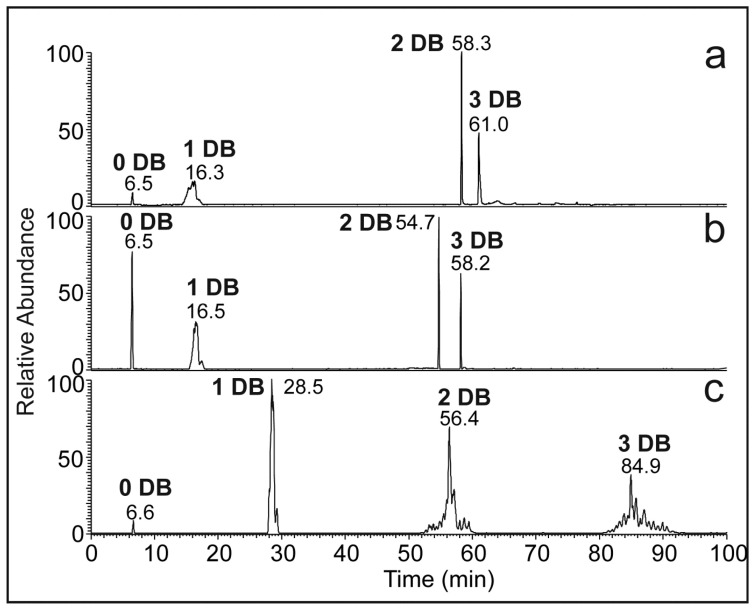
Ag-HPLC/APCI-MS chromatograms of *N. bullata* cuticular HCs recorded in the solvent system (**a**) I (hexane, acetonitrile, and 2-propanol; a linear increase in B at 0.556% B/min), (**b**) II (isooctane, acetonitrile, and 2-propanol; a linear increase in B at 0.556% B/min), and (**c**) III (hexane and toluene; a linear increase in B at 0.66% B/min).

The Ag-HPLC separation system was optimized using ChromSpher Lipids columns and gradients elution with mobile phases composed of hexane/2-propanol/acetonitrile (solvent system I), isooctane/2-propanol/acetonitrile (solvent system II), and hexane/toluene (solvent system III), see Table 1. The mobile phase systems were designed concerning their suitability for Ag-HPLC and APCI-MS detection of HCs [43,56,57]. Toluene and acetonitrile were assumed to interact predominantly with the silver ions, thus reducing the interactions with the double bonds. In contrast, methanol, 2-propanol, and acetone decreased the strength of the interactions with the silanol groups and other polar moieties [58]. Therefore, the composition of the mobile phase can be adjusted to achieve a high resolution of HC species differing in the number, configuration, and position of double bonds. Figure 1 shows Ag-HPLC/APCI-MS chromatograms of *N. bullata* HCs recorded in all three mobile phase systems. As expected, HCs eluted according to the increasing number of double bonds. The solvent systems I and II provided a similar pattern of peaks, and the retention times for saturated and monounsaturated HC were almost the same. Unsaturated HCs with two and three double bonds were strongly retained in the mobile phase containing hexane (system I). In the solvent systems I and II, di- and triunsaturated HCs eluted as sharp peaks, with no separation of the molecular species within these groups. The solvent system III (hexane/toluene) provided higher retention times for unsaturated HCs. Importantly, chromatographic separation of the molecular species within each group of unsaturated HCs was observed. The retention behavior of HCs was consistent with the previous study dealing with Ag-HPLC of *n*-alkadienes [32].

Earlier reports showed that the column temperature strongly influences chromatographic resolution in Ag-HPLC [36,56,58]. Therefore, the HCs separation in all three solvent systems was probed at 15 °C, 25 °C, and 35 °C (Table 2). The retention times of unsaturated HCs were reduced by increasing temperatures in all three solvent systems. Surprisingly, the effect of the temperature was the complete opposite compared to the ester lipids reported previously [36,56,58]. The temperature effects in Ag-HPLC are explained by the different temperature-dependent stabilities of the complexes formed between silver ions and analytes [58]. In addition to the interactions of the chain double-bond electrons, silver ion complexes can also be formed by the interactions of carbonyls in ester lipids. Therefore, we assume that the absence of an ester group is responsible for the observed temperature effect. The retention order in all solvent systems followed the number of double bonds. Unfortunately, the column temperature did not influence the chromatographic resolution of the compounds with the same number of double bonds too much (Supplementary Figure S1). No separation of the compounds with the same number of double bonds was observed in solvent systems I and II. The best separation of HCs in solvent system III was observed at 15 °C.

The effect of the steepness of the mobile phase gradient was investigated in the next step. In the solvent systems I and II, changing the gradient steepness (a linear increase in B in the range 1.220–0.278%/min) had no major effect on the chromatographic separation: HCs with the same number of double bonds coeluted. As regards the solvent system III, the chromatographic resolution was enhanced at slower gradients.

**Figure 2:**
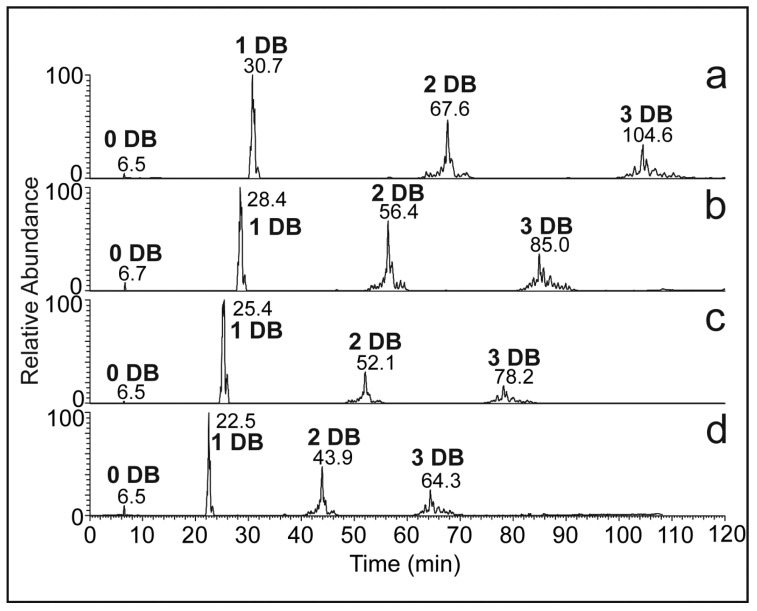
Ag-HPLC/APCI-MS chromatograms of *N. bullata* cuticular HCs recorded in the solvent system III. Linear increase in toluene and (B) in hexane (A) at (**a**) 0.50% B/min, (**b**) 0.66% B/min, (**c**) 0.75% B/min, and (**d**) 1.00% B/min. The column temperature was 15 °C.

Figure 2 shows Ag-HPLC chromatograms of *N. Bullata* HCs recorded using various gradient slopes (a linear increase in B at 1.00, 0.75, 0.66, and 0.50%/min) at 15 °C. The best separation was observed for the slowest gradient; further lowering the gradient slope would not be convenient because of long retention times, peak broadening, and decreased sensitivity.

**Figure 3:**
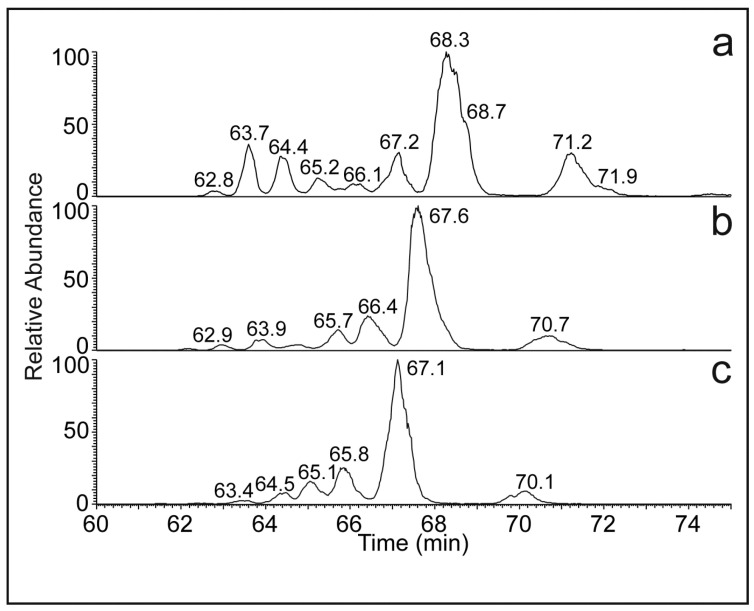
Chromatograms of diunsaturated HCs ([M + H]^+^) reconstructed for (**a**) *m*/*z* 545 (C_39_H_77_ and C39:2), (**b**) *m*/*z* 573 (C_41_H_81_ and C41:2), and (**c**) *m*/*z* 601 (C_43_H_85_ and C43:2). For the experimental conditions, see Figure 2a.

Figure 3 shows a chromatogram of *N. Bullata* HCs reconstructed for three *m*/*z* values corresponding to diunsaturated HCs. The peaks (mostly double-bond positional isomers) were separated almost to the baseline. Slight shifts in the retention times (cf. Figure 3a–c) indicate the separation of the aliphatic HCs according to the number of carbon atoms. The decrease in retention times with the number of carbon atoms can be rationalized by a normal phase separation mode, which partially occurs in Ag-HPLC systems. Similar behavior was previously observed for TGs, WEs, and FAMEs [35].

**Figure 4:**
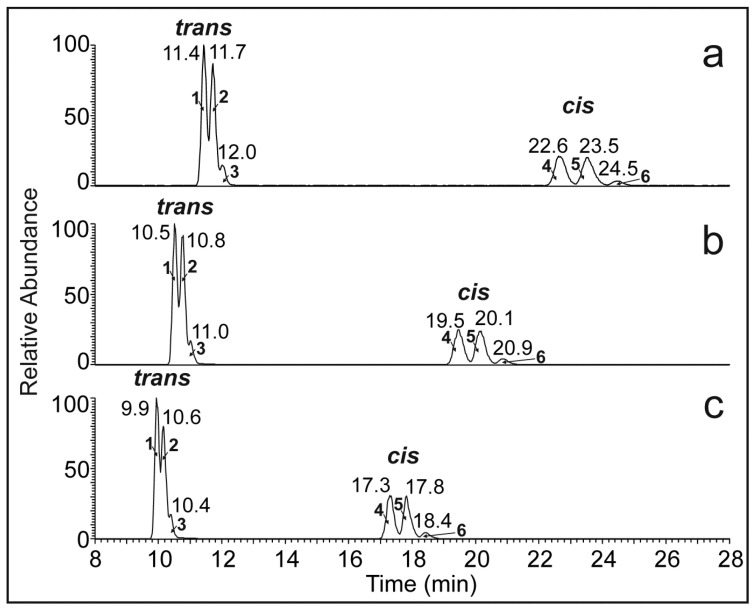
Ag-HPLC/APCI-MS chromatogram of HC standards recorded at the column temperature (**a**) 15 °C, (**b**) 25 °C, and (**c**) 35 °C. The peak identification: 1. 19*t*-C38:1, 2. 13*t*-C32:1, 3. 13*t*-C26:1, 4. 19*c*-C38:1, 5. 13*c*-C32:1, and 6. 13*c*-C26:1. For the experimental conditions, see Figure 2a.

**Figure 5:**
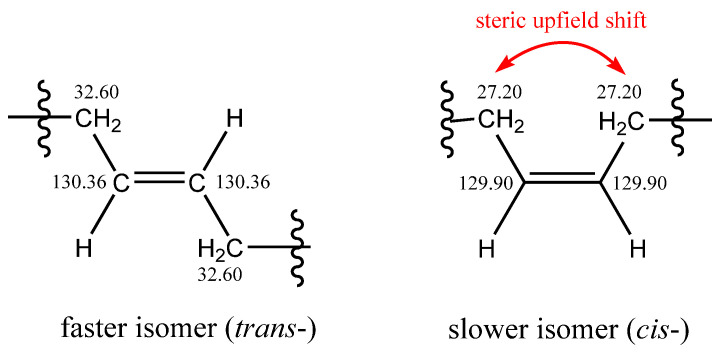
Carbon-13 chemical shifts of selected atoms and distinguishing between trans and cis isomers.

Cuticular HCs biosynthesized from fatty acids mostly contain cis double bonds. To investigate the effect of double-bond geometry, a synthetic mixture containing cis and trans isomers was used. The mixture of 13*c*-C26:1, 13*c*-C32:1, 19*c*-C38:1, 13*t*-C26:1, 13*t*-C32:1, and 19*t*-C38:1 was separated in solvent system III. Two clusters of peaks appeared in the chromatograms (Figure 4), indicating the separation of the cis/trans isomers. Excellent separation of cis/trans isomers of various lipids in Ag-HPLC is well known; trans isomers elute faster because their interactions with silver ions are less efficient due to a larger steric hindrance [35]. We used NMR to elucidate double-bond geometry in two 16-C32:1 isomers isolated by preparative Ag-HPLC. The NMR spectra of the isomers were similar and confirmed the molecule’s symmetry, resulting in half the number of NMR signals. The symmetry equivalence of olefinic protons made it impossible to use their mutual coupling to distinguish cis and trans isomers. Therefore, we used a difference in ^13^C chemical shifts of the allylic carbon atoms. The faster isomer showed an allylic carbon at 32.60 ppm, while the slower one was at 27.20 ppm. The −5.4 ppm shift in the slower isomer can be explained by the steric upfield shift between two methylene groups in the cis isomer. At the same time, such an effect is absent in the faster isomer with a trans double-bond configuration (see Figure 5). In this way, we confirmed that the trans isomer eluted faster. The column temperature moderately affected the chromatographic separation of species within the trans and cis isomer groups; the best resolution was observed at 15 °C (cf. Figure 4a–c).

To conclude the chromatographic experiments, two ChromSpher Lipids columns in series at 15 °C and a hexane/toluene gradient with a toluene increase rate of 0.5%/min provided the best separation conditions for unsaturated HCs. Compared to other solvent systems tested (systems I and II), the optimized method showed superior reproducibility of the retention times and required the shortest equilibration times. The excellent miscibility of toluene and hexane positively affected the method’s robustness. The RSDs of the retention times were 1.1% at the most for the HPLC runs made on the same day (n = 3) and up to 2.3% for measurements made within a month (n = 6).

### 2.2. The APCI-MS Detection of HCs Separated by HPLC

A range of ions can be observed in APCI spectra of hydrocarbons, including M^+^, [M + H]^+^, [M − H]^+^, solvent-related adducts, and fragments. The molecular ions M^+^ are likely formed by electron transfer reactions with ionized nitrogen species, while proton transfers from strong Brønsted acids such as N_2_H^+^ and H_3_O^+^ create [M + H]^+^ [50]. The formation of [M − H]^+^ from saturated hydrocarbons was recently rationalized by eliminating molecular hydrogen from protonated molecules [50]. Since the proton transfer reactions are highly exothermic, fragment ions are formed. Solvent-related adducts are products of chemical ionization processes in the ion source, and their nature reflects the mobile phase composition.

**Figure 6:**
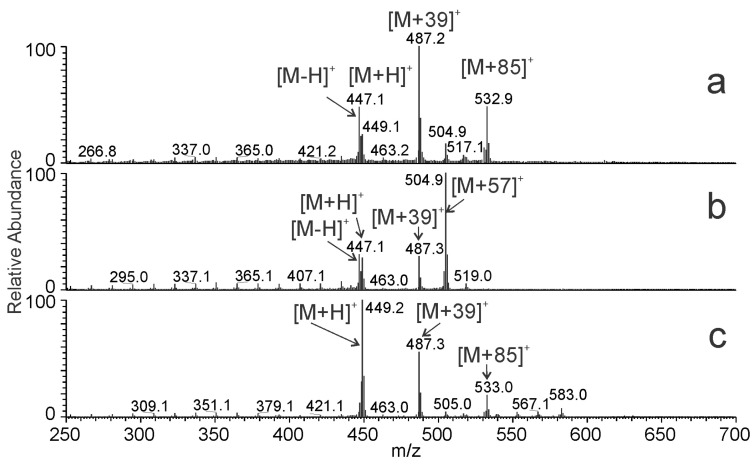
APCI-MS spectra of 13*c*-C32:1 recorded in (**a**) hexane/acetonitrile/2-propanol (99.95/0.025/0.025, by vol.), (**b**) isooctane/acetonitrile/2-propanol (99.95/0.025/0.025, by vol.), and (**c**) hexane/toluene (95/5).

**Figure 7:**
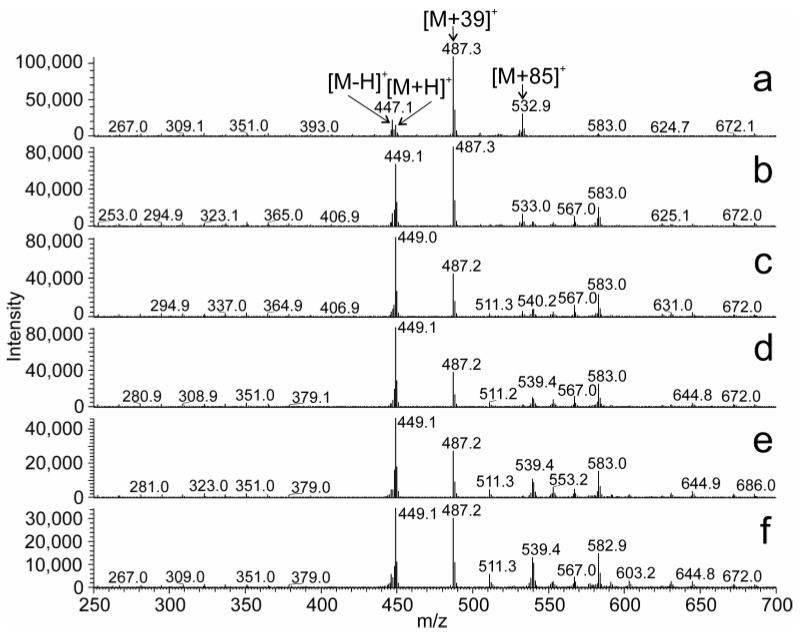
APCI-MS spectra of a mixture of 16c-C32:1 and 16t-C32:1 recorded in (**a**) hexane, (**b**) hexane/toluene (80/20, by vol.), (**c**) hexane/toluene (60/40, by vol.), (**d**) hexane/toluene (40/60, by vol.), (**e**) hexane/toluene (20/80, by vol.), and (**f**) toluene at the flowrate 1 mL/min.

The effect of various solvents on the APCI spectra was exemplified for 13*c*-C32:1. The mass spectra of this compound recorded in the solvent systems I, II, and III at the corresponding retention times are shown in Figure 6. In the solvent system I (Figure 6a), 13*c*-C32:1 eluted in hexane/acetonitrile/2-propanol (99.95/0.025/0.025, by vol.). The most abundant ion [M + 39]^+^ was likely a molecular adduct with C_3_H_3_^+^, whereas [M + 85]^+^ corresponded to an adduct with C_6_H_13_^+^ ([hexane − H]^+^). The relative intensity of [M − H]^+^ was higher than that of [M + H]^+^. In the solvent system II (Figure 6b), 13*c*-C32:1 eluted in isooctane/acetonitrile/2-propanol (99.95/0.025/0.025, by vol.), and the most abundant signal was [M + 57]^+^. This ion was likely an adduct with C_4_H_9_^+^ formed from isooctane (2,2,4-trimethylpentane). The intensities of [M − H]^+^ and [M + H]^+^ were almost equal and roughly half of [M + 57]^+^. In the solvent system III (Figure 6c), 13c-C32:1 was eluted in 95% hexane and 5% toluene. The relative intensity of the protonated molecule was high, and [M + H]^+^ was the spectrum base peak. The absolute intensity of the [M + H]^+^ was the highest among the systems tested. In the next experiments, we recorded APCI spectra of 16*c*-C32:1 dissolved in mobile phases differing by their proportions of hexane and toluene (Figure 7). In 100% hexane, 16*c*-C32:1 yielded mostly [M + 39]^+^, and the intensity of [M + H]^+^ was rather small. The intensity of [M + H]^+^ increased with the increasing concentration of toluene in the mobile phase and reached it maximum at 40–60% toluene. A further rise in the toluene concentration caused a decrease in the [M + H]^+^ peak. Ion source fragments of the aliphatic chain were also observed for all solvent systems. The intensities of the fragments were up to 8% of the base peak.

**Figure 8:**
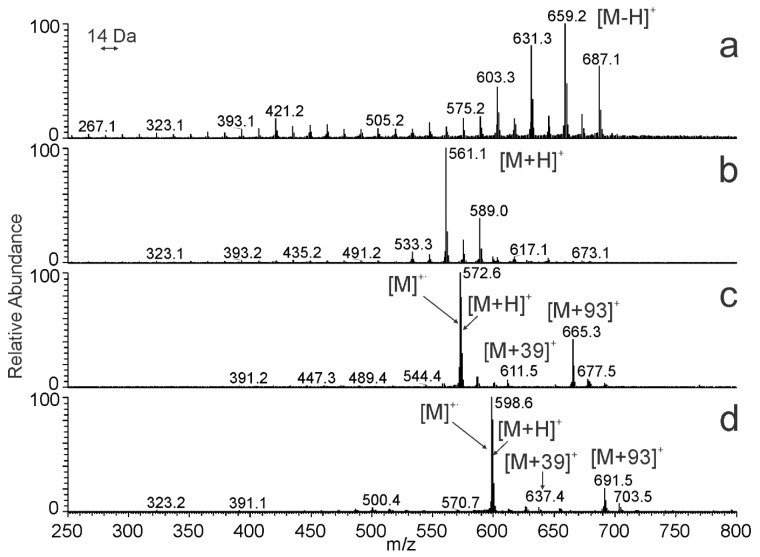
APCI-MS spectra of *N. bullata* cuticular HCs having (**a**) none, (**b**) one, (**c**) two, and (**d**) three doubles averaged across the most abundant peaks in the HPLC analysis at the retention times (**a**) 6.4–6.5 min, (**b**) 30.7–30.8 min, (**c**) 67.6–67.7 min, and (**d**) 104.4–104.5 min. For the experimental conditions, see Figure 2a.

The ions formed from HCs in APCI also depended on the number of double bonds in their molecules. The number of double bonds significantly affects the detection sensitivity in many neutral lipids [59,60]. APCI spectra taken across chromatographic peaks thus reflected a combined effect of mobile phase composition and hydrocarbon structure. Figure 8 shows spectra taken across the most abundant chromatographic peaks in the *N. bullata* sample. Coeluting saturated HCs (Figure 8a) provided abundant signals of [M − H]^+^, which is in agreement with previous observations [46,50,61].

In contrast to saturated species, unsaturated HCs were detected as protonated molecules [M + H]^+^ (Figure 8b–d) [46,53]. In addition to the protonated molecules, di- and triunsaturated HCs eluting at high concentrations of toluene provided abundant radical cations [M]^+·^ and also adducts with protonated toluene [M + 93]^+^ (Figure 8c,d). As evident from previous paragraphs, interpreting the APCI spectra of HCs is not always straightforward. However, the spectra give enough information for a reliable assignment of the molecular weight, number of carbons, and number of double bonds.

### 2.3. Cuticular HCs of N. bullata

The optimized Ag-HPLC/MS method made it possible to detect 136 cuticular HCs in *N. bullata* and characterize them by the number of carbons, double bonds, and double-bond geometry (Table 3). The relative peak areas were used to estimate the proportions of HCs. It is important to note that the relative peak areas do not correctly reflect real abundances, mainly because of large differences between the response factors of analytes with different numbers of double bonds. As discussed previously [59,60], APCI-MS tends to provide a higher response for unsaturated lipids and thus underestimates saturated species. The relative peak areas are reasonable estimates within the groups of compounds with the same degree of unsaturation. The chain length also influences the detection sensitivity, but its effect is less important [59,62].

Saturated HCs eluted close to the column dead volume. Chromatograms reconstructed for [M − H]^+^ ions showed a gradual decrease in the retention time with the number of carbon atoms, indicating a normal phase separation mechanism (Table 3). Nevertheless, retention times were similar; all saturated HCs eluted within a ca. 7-second interval. Saturated HCs contained 26–49 carbon atoms, with C30:0, C45:0, and C47:0 being the most abundant species. Monounsaturated HCs were better resolved; they contained 27–47 carbons, with C37:1, C39:1, C40:1, and C42:1 providing the highest signals. The cis-C38:1 and cis-40:1 were detected at two different retention times, likely because of different double-bond positions. Their cis double-bond geometry was deduced from their retention times closely matching synthetically prepared standards (cf. Figure 4). Many di- and triunsaturated HCs, including isomers found in several chromatographically separated peaks, were detected. Diunsaturated HCs contained 37–45 carbons and cis double bonds; C39:2, C41:2, and C42:2 were the most abundantly present (Figure 3). Interestingly, minor HC peaks with a trans double bond(s) were observed (trans-monounsaturated HCs at R_t_ 21.3–22.7; trans-diunsaturated HCs at R_t_ 50–55.5 min (Figure S2) and trans-triunsaturated HCs at R_t_ 84.1–89.9 min). Because of their low abundance (below the threshold), they are not listed in Table 3. The overall ratio of HCs with cis and trans (at least one) double bonds estimated from the peak areas was ca 1000:1. HCs with trans double bonds are extremely rare in insect cuticular HCs [2,3,4].

The overall number of HCs detected in *N. bullata* by Ag-HPLC/APCI-MS (136) was much higher than previously reported for GC [7,54,55] or MALDI-TOF MS [23]. Two reasons for more successful detection by HPLC/MS can be proposed: (a) an efficient chromatographic separation of unsaturated HCs in HPLC provides good resolution for isomeric HCs, and (b) the detection of nonvolatile (or degradable) long-chain HCs is not amenable to GC. Earlier papers based on GC reported 32 saturated HCs with 25–32 carbons, both straight-chain and branched [54,55]. The presence of heavier HCs was evident, but their characterization has not been performed for their low concentration. Later, HTGC/MS made it possible to detect almost 50 chromatographically separated peaks representing C25-C46 HCs. The nonpolar GC column provided limited selectivity for unsaturated HC isomers. It can be demonstrated by two isomers of C41:2 detected by HTGC/MS [7] and seven isomers of the same HCs resolved by Ag-HPLC (Table 3). MALDI-MS spectrum of HCs from *N. bullata* [7,23] showed ca 60 compounds with 25–49 carbons and 0–3 double bonds. The relatively low number of detected compounds resulted from detecting isobaric isomers at the same *m*/*z* values. On the other hand, summing up signals of very low abundant isomers with the same mass made it possible to detect them by MALDI-MS. This is probably why Ag-HPLC/APCI-MS detected unsaturated HCs up to C47, but MALDI-MS saw a little bit further and additionally detected C48 and C49 HCs. Hydrocarbons differing by one double bond can be difficult to distinguish in a MALDI spectrum because the signal of the more saturated hydrocarbon overlaps with the second isotopic peak of the less saturated specie.

### 2.4. Cuticular HCs of P. americana

Compared to *N. bullata*, the cuticular HCs from the cockroach *P. americana* were less complex. Ag-HPLC/APCI-MS disclosed 44 hydrocarbons (Table 4), 31 of which were saturated, 7 monounsaturated, 3 diunsaturated, and 3 triunsaturated. Saturated and monounsaturated species were eluted in narrow chromatographic zones; the signal of diunsaturated HCs was rather broad, which is likely due to a high abundance of C27:2 (Figures S3 and S4). The [M − H]^+^ peaks in the spectrum of saturated HCs spanned over the entire *m*/*z* range, indicating the presence of HCs C25:0–C63:0. The HCs C26:0, C28:0, C40:0, C:42:0, C44:0, and C45:0 were the most abundant saturated species. The newly reported HCs 46:0–63:0 were detected at low concentration levels (around 1%). The monounsaturated HCs consisted of C27:1, C40:1, C41:1, C43:1, and C45:1, whereas C41:1 and C43:1 formed 91% of all monounsaturated HCs. Three different hydrocarbons corresponding to 27:1 were found. Diunsaturated HCs contained 27–29 carbons, and the major HC in this group was cis/cis-C27:2. As in the case of *N. bullata*, traces of HCs with one or two trans double bonds were detected at lower retention times (Figure S5). Finally, the presence of triunsaturated HCs C41:3, C43:3, and C45:3 was observed at R_t_ 117–119 min. As for *N. bullata*, Ag-HPLC/APCI-MS made it possible to detect significantly more cuticular HCs than the other methods. Existing literature based on GC reports 10 [63], 11 [64], or 27 [65] cuticular HCs in *P. americana,* which is about half of the HCs described in this work. MALDI MS made it possible to detect approximately 17 peaks of HCs [23]. In all reports, including this one, C27:2 was the major component of the cockroach’s cuticular HCs.

## 3. Materials and Methods

### 3.1. Chemicals and Materials

Mobile phase components acetonitrile (LC/MS grade), 2-propanol (LC/MS grade), toluene (HPLC grade), and *n*-hexane (HPLC grade) were all obtained from Sigma-Aldrich (St. Louis, MO, USA), which, along with 2,2,4-trimethylpentane (isooctane, HPLC grade Lab-Scan, Lach-Ner, Neratovice, Czech Republic), were dried over a molecular sieve (4 Å, pellets of 3.2 mm in diameter, Sigma-Aldrich). Chloroform and diethyl ether were distilled in glass from analytical-grade solvents.

Standards of monounsaturated HCs were synthesized by metathesis as follows: Grubbs’ catalyst, 1st generation (1 mg, 0.0012 mmol, Sigma-Aldrich), was added to a mixture of eicos-1-ene (50.5 mg, 0.18 mmol; Sigma-Aldrich) and tetradec-1-ene (35.3 mg, 0.18 mmol; Sigma-Aldrich), and the mixture was heated for 16 h under argon at 40 °C in an oil bath. The reaction mixture was suspended in hexane, filtered through a silica gel plug (500 mg), and the products were eluted from the plug using hexane (3 × 3 mL). Hexane was evaporated on a rotary vacuum evaporator, and the resulting product was dried by an oil pump vacuum for 1 h to yield 27 mg. The composition of the product was determined by GC/MS based on the peak areas in the total ion current chromatogram; the product contained (13*E*)-hexacos-13-ene (13*t*-C26:1; 20%), (13*E*)-dotriacont-13-ene (13*t*-C32:1; 36%), (19*E*)-octatriacont-13-ene (19*c*-C38:1; 14%), (13*Z*)-hexacos-13-ene (13*c*-C26:1; 9%), (13*Z*)-dotriacont-13-ene (13*c*-C32:1; 16%), and (19*Z*)-octatriacont-13-ene (19*c*-C38; 5%). The stock solution of the hydrocarbon mixture was prepared in chloroform at a concentration of 5 mg/mL. A mixture of cis and trans isomers of dotriacont-16-ene was prepared by the same procedure from heptadec-1-ene (78 mg, 0.32 mmol; Sigma-Aldrich). The dotriacont-16-ene isomers were then isolated from the mixture using Ag-HPLC (Section 3.2, solvent system III).

Cuticular HCs were isolated from flies and cockroaches as follows: gray flesh flies, *Neobellieria bullata* (Parker, 1916) (*Diptera: Sarcophagidae*), and cockroaches, *Periplaneta americana* (Linnaeus, 1758) (*Blattaria: Blattidae*), were from the laboratory breeding of the Institute of Organic Chemistry and Biochemistry, Prague. Adult flies were immobilized at −18 °C and placed for 12 h in a desiccator to remove surface moisture. HCs were extracted with chloroform (3 × 50 mL, 3 min). The volume of solvent was reduced in a vacuum evaporator. Cuticular HCs were isolated by a preparative TLC on pre-cleaned glass plates (36 × 76 mm) coated with Silica gel 60 G (Merck KGaA, Darmstadt, Germany) using hexane as a mobile phase [21]. The TLC spots were visualized by spraying rhodamine 6G solution (0.05% in ethanol), which was then scraped off the plates and extracted with diethyl ether. HCs were obtained from the upper part of the TLC plates (R_F_ 0.5–1.0). Diethyl ether was removed under a stream of argon. Cuticular HCs were dissolved in chloroform to a concentration of 50 mg/mL (stock solution) and diluted before the analysis in chloroform to 10 mg/mL.

### 3.2. HPLC/APCI-MS

The liquid chromatograph consisted of an Accela autosampler, a Rheos Allegro UHPLC pump, and an LCQ Fleet ion-trap mass spectrometer. The system was controlled by Xcalibur software (all by Thermo Fisher Scientific, San Jose, CA, USA). Two Ag-HPLC ChromSpher Lipids columns (250 × 4.6 mm, particle size: 5 μm; Varian, Palo Alto, CA, USA) were connected in series. A column thermostat, which is a part of the autosampler, maintained the column temperature at 15 °C, 25 °C, or 35 °C. Three mobile phase systems were tested (Table 1); the gradient program started with 100% of solvent A, and the proportion of solvent B increased linearly during analysis. The flow rate of the mobile phase was 1.0 mL/min, and the injected volume of the samples was 3 µL or 5 µL. The columns were conditioned with 100% of A (100 µL/min) for at least 12 h before the first analysis and equilibrated for 1 h (solvent systems I and II) or 30 min (solvent system III) between injections. The APCI vaporizer and heated capillary temperatures were set to 350 °C and 150 °C, respectively; the corona discharge current was 3.5 µA. Nitrogen served as the sheath and auxiliary gas at a flow rate of 60 and 45 arbitrary units, respectively. The mass spectra of the positively charged ions were recorded from *m*/*z* 200 or 300 to *m*/*z* 900. The peak areas in the chromatograms reconstructed for the sum of [M − H]^+^, [M]^+·^, and [M + H]^+^ ions were used for estimating the relative concentrations of HCs.

### 3.3. NMR

The NMR spectra were acquired with spectrometer Bruker AVANCE 500 (^1^H at 500.1 MHz and ^13^C at 125.8 MHz) that was equipped with a cryoprobe in CDCl_3_ at 25 °C. Homonuclear 2D-NMR spectra (H,H-COSY) and heteronuclear 2D-NMR spectra (H,C-HSQC) were used for a partial structural assignment of proton and carbon signals and determination of double-bond configuration in olefinic isomers.

## 4. Conclusions

A new method for analyzing cuticular HCs was developed. Chromatographic separation according to the number and geometry of double bonds was achieved on an LC column containing silver ions. The mobile phase composition and gradient program were optimized, and the best results were achieved in a solvent system consisting of hexane and toluene. The optimized HPLC conditions made it possible to separate groups of saturated, mono-, di-, and triunsaturated HCs. Within the groups, a partial separation according to the chain length occurred. The chromatographic resolution increased with the number of double bonds. The great advantage of this method is the excellent separation of geometrical isomers, which allows cis and trans isomers to be resolved. Despite the formation of various structure- and solvent-dependent ions, APCI-MS detection made it possible to determine the molecular weight and, thus, the number of carbon atoms and double bonds. Ag-HPLC/APCI-MS efficiently detected long-chain HCs, which are difficult (or impossible) to analyze by GC/MS. Compared to MALDI-TOF, the main advantage of Ag-HPLC/APCI-MS is the ability to distinguish isomers and separate species differing by two hydrogens, i.e., a double bond. The increased sensitivity to HCs with one or more double bonds is another advantage of the method.

Further research is needed to find out how tandem mass spectrometry can be used to obtain more detailed information about the HCs structures (e.g., branching sites and positions of double bonds). Ag-HPLC/APCI-MS has great potential to become a new tool for studying cuticular HCs in chemical ecology. The method can discover new HCs that mediate various insect behaviors and cannot be detected by the existing analytical tools.

## Figures and Tables

**Table 1:** Mobile phase systems for Ag-HPLC/APCI-MS of HCs.

Solvent System	Solvent A (vol.%)	Solvent B (vol.%)
I	hexane/acetonitrile/2-propanol (99.95/0.025/0.025)	hexane/acetonitrile/2-propanol (98.0/1.0/1.0)
II	isooctane/acetonitrile/2-propanol (99.95/0.025/0.025)	isooctane/acetonitrile/2-propanol (98.0/1.0/1.0)
III	Hexane	Toluene

**Table 2:** Retention times of the most abundant peaks in the chromatograms of *N. bullata* at various column temperatures.

Solvent System	T (°C)	Retention Time of the Most Abundant Peak (min)			
		Saturated	Monounsaturated	Diunsaturated	Triunsaturated
I	15	6.7	28.1	66.1	66.1
	25	6.5	16.0	61.0	66.5
	35	6.5	16.5	54.5	60.1
II	15	6.5	16.5	61.1	64.1
	25	6.5	16.5	54.7	58.2
	35	6.6	17.0	52.2	56.2
III	15	6.5	22.9	74.1	130.1
	25	6.4	18.9	65.3	115.4
	35	6.3	17.9	59.3	102.5

**Table 3:** Cuticular HCs of *N. bullata* identified by Ag-HPLC/APCI-MS.

Peak No.	Rt (min)	CN:DB ^1^	Relative Peak Area (%) ^2^	Peak No.	Rt (min)	CN:DB ^1^	Relative Peak Area (%) ^2^
0 double bond(s)							1.3
1	6.45	49:0	5.9	13	6.50	37:0	1.2
2	6.45	48:0	1.4	14	6.50	36:0	1.1
3	6.48	47:0	11.3	15	6.52	35:0	2.0
4	6.48	46:0	2.0	16	6.52	34:0	1.6
5	6.50	45:0	11.3	17	6.54	33:0	5.4
6	6.50	44:0	2.0	18	6.54	32:0	6.0
7	6.50	43:0	7.1	19	6.56	31:0	5.4
8	6.50	42:0	1.9	20	6.56	30:0	13.1
9	6.50	41:0	2.4	21	6.56	29:0	4.8
10	6.50	40:0	1.3	22	6.56	28:0	4.8
11	6.50	39:0	1.7	23	6.56	27:0	2.9
12	6.50	38:0	1.2	24	6.56	26:0	2.3
1 double bond(s)							37.9
25	30.28	47:1	3.9	37	31.17	37:1	11.8
26	30.34	46:1	0.6	38	31.19	36:1	2.4
27	30.36	45:1	5.8	39	31.30	35:1	1.6
28	30.50	44:1	0.8	40	31.38	34:1	0.8
29	30.50	43:1	5.1	41	31.46	33:1	0.5
30	30.67	42:1	11.7	42	31.46	32:1	1.2
31	30.73	40:1	10.7	43	31.61	31:1	2.0
32	30.86	41:1	4.1	44	31.69	30:1	0.4
33	30.86	40:1	8.7	45	31.73	28:1	0.4
34	30.93	38:1	7.2	46	31.77	29:1	2.1
35	30.95	39:1	12.1	47	31.92	27:1	1.3
36	31.04	38:1	5.0				
2 double bond(s)							43.1
48	55.38	45:2	0.6	73	66.48	45:2	1.7
49	62.18	41:2	0.1	74	66.82	44:2	2.3
50	62.77	39:2	0.2	75	66.84	40:2	0.2
51	62.93	41:2	0.4	76	67.12	43:2	8.0
52	63.53	43:2	0.1	77	67.16	39:2	1.7
53	63.60	39:2	1.6	78	67.45	37:2	0.1
54	63.75	45:2	0.2	79	67.45	42:2	3.1
55	63.91	41:2	1.1	80	67.63	41:2	22.8
56	64.25	37:2	0.2	81	67.97	40:2	1.7
57	64.34	43:2	0.3	82	68.27	39:2	10.4
58	64.36	39:2	1.4	83	68.47	38:2	0.1
59	64.51	45:2	0.4	84	68.81	38:2	0.1
60	64.80	41:2	0.4	85	68.85	46:2	0.6
61	65.03	37:2	0.3	86	69.12	37:2	0.8
62	65.05	43:2	0.9	87	69.43	45:2	0.2
63	65.07	46:2	0.3	88	69.64	44:2	1.7
64	65.22	39:2	0.5	89	70.13	43:2	0.8
65	65.55	44:2	0.5	90	70.37	42:2	18.1
66	65.71	37:2	0.1	91	70.73	41:2	2.5
67	65.71	41:2	1.8	92	70.86	40:2	0.4
68	65.86	43:2	1.7	93	71.23	39:2	2.6
69	66.17	39:2	0.4	94	71.32	40:2	0.6
70	66.26	42:2	0.6	95	71.74	38:2	0.2
71	66.26	46:2	1.0	96	71.90	37:2	0.2
72	66.42	41:2	3.9				
3 double bond(s)							17.7
97	99.90	47:3	0.6	117	106.23	43:3	1.6
98	100.73	47:3	0.6	118	106.83	41:3	13.9
99	101.33	45:3	1.3	119	106.97	45:3	1.9
100	101.53	47:3	3.0	120	107.30	49:3	0.4
101	102.12	45:3	1.7	121	107.52	43:3	1.9
102	102.58	47:3	0.9	122	107.86	41:3	2.6
103	103.01	45:3	7.5	123	107.90	45:3	0.8
104	103.5	43:3	0.9	124	108.60	43:3	3.9
105	103.69	44:3	0.6	125	108.94	39:3	0.4
106	103.84	45:3	4.2	126	108.97	41:3	0.5
107	104.44	47:3	0.4	127	109.43	39:3	0.3
108	104.55	43:3	22.0	128	109.52	43:3	1.4
109	104.55	44:3	0.4	129	110.31	41:3	4.2
110	104.94	45:3	1.2	130	110.77	43:3	0.4
111	105.10	41:3	0.6	131	111.37	41:3	2.0
112	105.18	42:3	0.6	132	112.33	41:3	0.9
113	105.20	43:3	13.0	133	113.05	39:3	0.7
114	105.49	47:3	0.7	134	113.76	41:3	0.3
115	105.92	45:3	0.5	135	114.17	39:3	0.5
116	106.03	42:3	0.5	136	114.21	43:3	0.2

^1^ CN:DB = Carbon number: Double bonds. ^2^ The relative peak area is a fairly good estimate of the relative abundances within the groups of hydrocarbons with the same number of double bonds; it cannot be used for comparing hydrocarbons differing by the number of double bonds because the APCI-MS response tends to increase with the number of double bonds.

**Table 4:** Cuticular HCs of *P. americana* identified by Ag-HPLC/APCI-MS.

Peak No.	Rt (min)	CN:DB ^1^	Relative Peak Area (%) ^2^	Peak No.	Rt (min)	CN:DB ^1^	Relative Peak Area (%) ^2^
0 double bond(s)							2.5
1	6.47	63:0	1.4	17	6.52	47:0	1.8
3	6.49	62:0	1.0	18	6.52	46:0	2.5
4	6.49	61:0	0.8	19	6.52	45:0	4.3
5	6.49	60:0	1.2	20	6.52	44:0	17.8
6	6.50	59:0	0.8	21	6.54	43:0	6.3
7	6.50	58:0	1.1	22	6.54	42:0	25.0
7	6.50	57:0	0.9	23	6.54	41:0	2.8
8	6.50	56:0	1.2	24	6.54	40:0	4.4
9	6.51	55:0	0.8	25	6.55	39:0	0.9
10	6.51	54:0	1.1	26	6.58	30:0	0.7
11	6.51	53:0	0.7	27	6.59	29:0	6.6
12	6.51	52:0	1.3	38	6.59	28:0	1.4
13	6.51	51:0	1.0	29	6.59	27:0	1.5
14	6.52	50:0	1.6	33	6.59	26:0	5.2
15	6.52	49:0	1.3	31	6.59	25:0	1.0
16	6.52	48:0	1.8				
1 double bond(s)							17.0
32	29.11	43:1	40.8	36	29.90	27:1	0.3
33	29.14	27:1	0.4	37	29.98	45:1	2.3
34	29.23	41:1	50.6	38	30.20	27:1	4.0
35	29.24	40:1	1.6				
2 double bond(s)							65.3
39	66.14	29:2	0.4	41	66.38	27:2	99.3
40	66.19	28:2	0.3				
3 double bond(s)							15.2
42	117.31	45:3	3.1	44	118.97	41:3	25.3
43	118.01	43:3	71.6				

^1^ CN:DB = Carbon number: Double bonds. ^2^ The relative peak area is a fairly good estimate of the relative abundances within the groups of hydrocarbons with the same number of double bonds; it cannot be used for comparing hydrocarbons differing by the number of double bonds because the APCI-MS response tends to increase with the number of double bonds.

## Data Availability

The data presented in this study are available on request from the corresponding author.

## Supplementary Materials

The following supporting information can be downloaded at https://www.mdpi.com/article/10.3390/molecules28093794/s1, Figure S1: Chromatograms of *N. bullata* cuticular HCs; Figure S2: Chromatogram and mass spectra of diunsaturated HCs from *N. bullata*; Figure S3: Chromatogram of *P. americana* cuticular HCs; Figure S4: APCI spectra of *P. americana* cuticular HCs; Figure S5: Chromatogram and mass spectra of diunsaturated HCs from *P. americana.*
