# Supplementary material for: Silver Ion High-Performance Liquid Chromatography—Atmospheric Pressure Chemical Ionization Mass Spectrometry: A Tool for Analyzing Cuticular Hydrocarbons
Source: Molecules. 2023 Apr 28;28(9):3794. doi: 10.3390/molecules28093794 (PMC10179885; doi:10.3390/molecules28093794)
Supplement: Supplementary file 1 [file molecules-28-03794-s001.zip › molecules-2333407-supplementary.pdf]

Supplementary Material

Silver Ion High-Performance Liquid Chromatography—Atmospheric Pressure Chemical Ionization Mass Spectrometry: A Tool for Analyzing Cuticular Hydrocarbons

Vladimír Vrkoslav[1], Petra Horká[1,2], Jindřich Jindřich[3], Miloš Buděšínský[1], Josef Cvačka[1,2*]

1. *Institute of Organic Chemistry and Biochemistry of the Czech Academy of Sciences, Flemingovo Náměstí 542/2, 160 00 Prague, Czech Republic*

2. *Department of Analytical Chemistry, Faculty of Science, Charles University, Hlavova 2030/8, 128 00 Prague, Czech Republic*

3. *Department of Organic Chemistry, Faculty of Science, Charles University, Hlavova 2030/8, 128 00 Prague, Czech Republic*

** Correspondence: josef.cvacka@uochb.cas.cz*

List of content:

Figure S1 - Chromatograms of *N. bullata* cuticular HCs 2

Figure S2 - Chromatogram and mass spectra of diunsaturated HCs from *N. bullata* 3

Figure S3 - Chromatogram of *P. americana* cuticular HCs 4

Figure S4 - APCI spectra of *P. americana* cuticular HCs 5

Figure S5 - Chromatogram and mass spectra of diunsaturated HCs from *P. americana* . . . 6

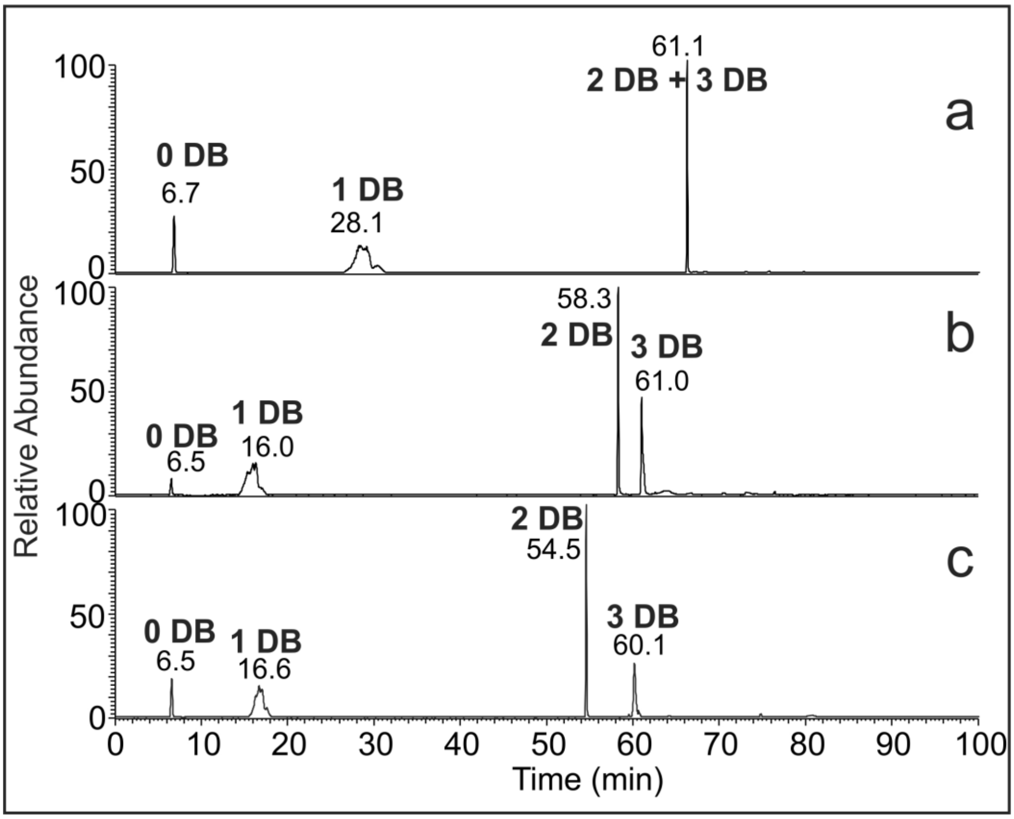

Figure S1. Chromatograms of *N. bullata* cuticular HCs at (a) 15°C, (b) 25°C, and (c) 35°C. The sample was separated in the solvent system I with a linear increase of toluene (B) in hexane (A) at 0.556 % B/min.

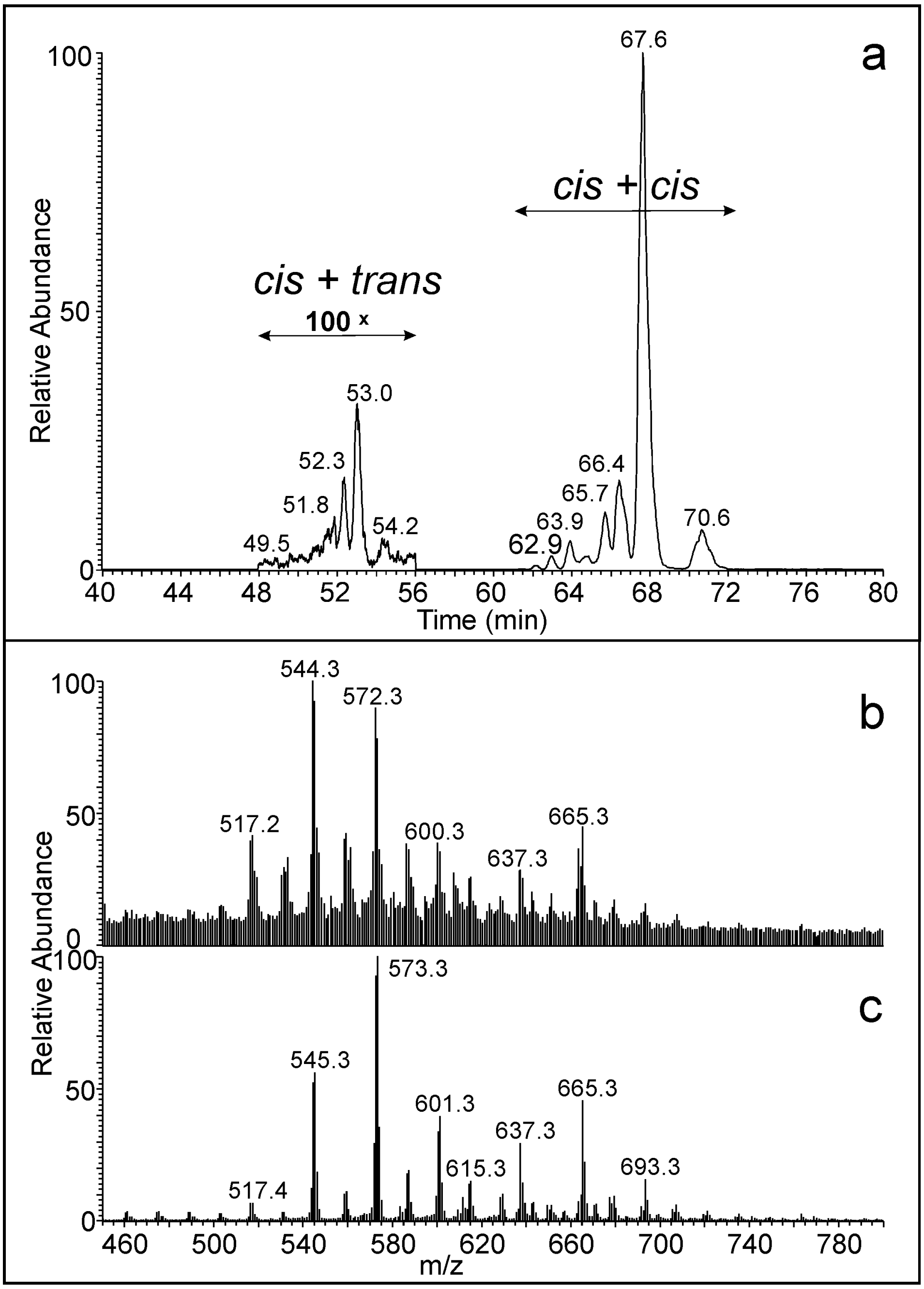

Figure S2. (a) Basepeak chromatogram of diunsaturated HCs from *N. bullata*, (b) APCI-MS spectrum averaged within R_t 50.0-55.5 min (one trans double bond, one cis), and (c) APCI-MS spectrum averaged within R_t 61.4-71.8 min (two cis double bonds). The sample was separated in the solvent system III with a linear increase of toluene (B) in hexane (A) at 0.50 % B/min. The column temperature was 15°C.

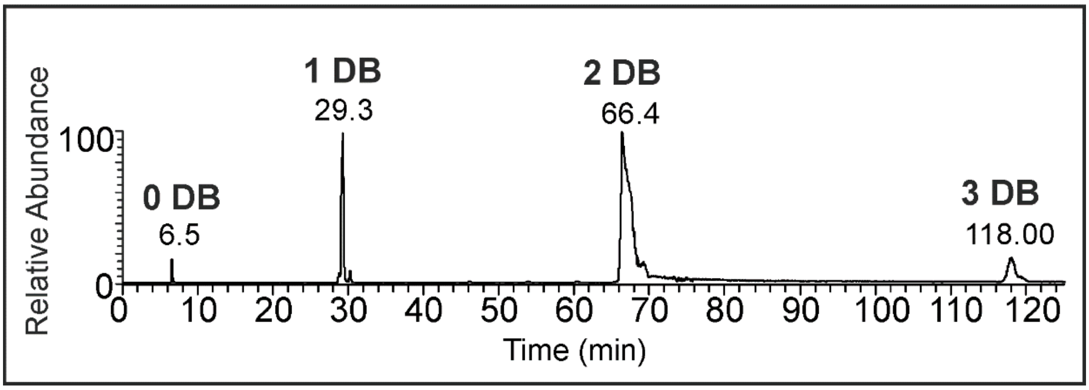

Figure S3. Ag-HPLC/APCI-MS chromatogram of *P. americana* cuticular HCs. The sample was separated in the solvent system III with a linear increase of toluene (B) in hexane (A) at 0.50 % B/min. The column temperature was 15°C.

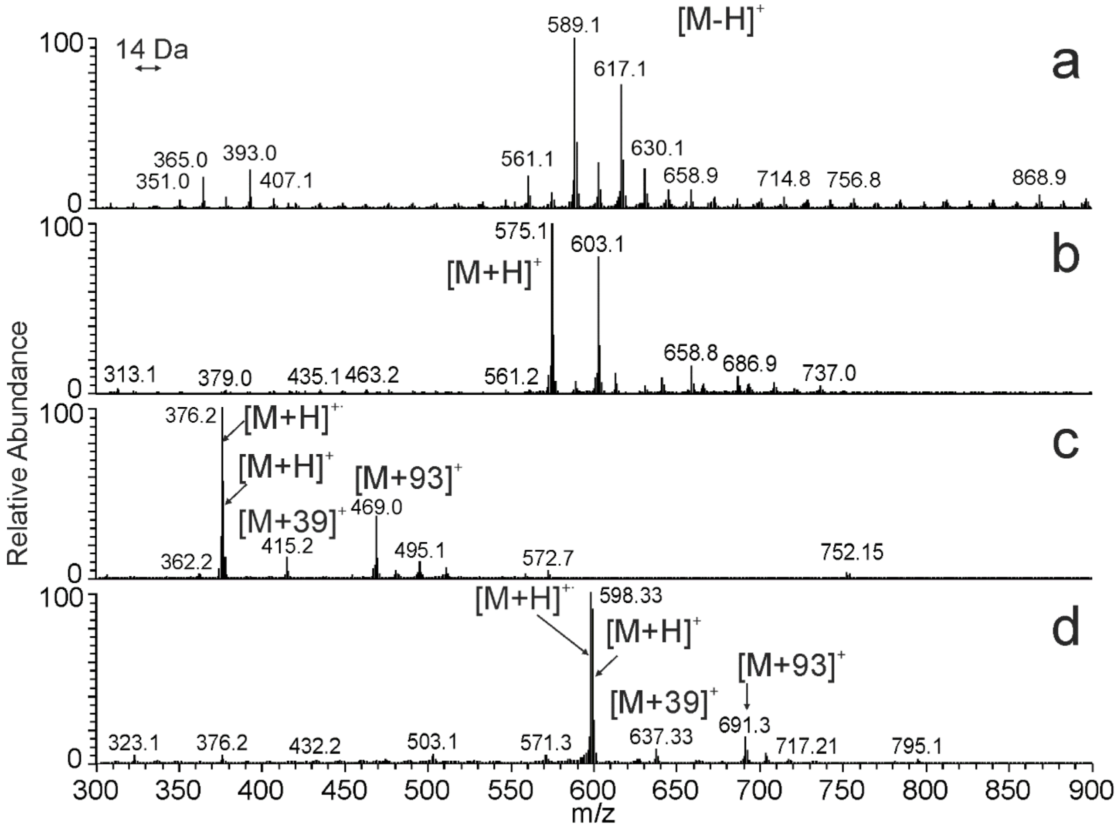

Figure S4. APCI spectra of *P. americana* cuticular HCs with a) 0, b) 1, c) 2, and d) 3 double bonds. The spectra were taken from the most abundant chromatographic peaks, a) 6.1-6.8 min, b) 29.0-29.4 min, c) 67.9-68.9 min, and d) 117.4-118.2 min. The sample was separated in the solvent system III with a linear increase of toluene (B) in hexane (A) at 0.50 % B/min. The column temperature was 15°C (Figure S3).

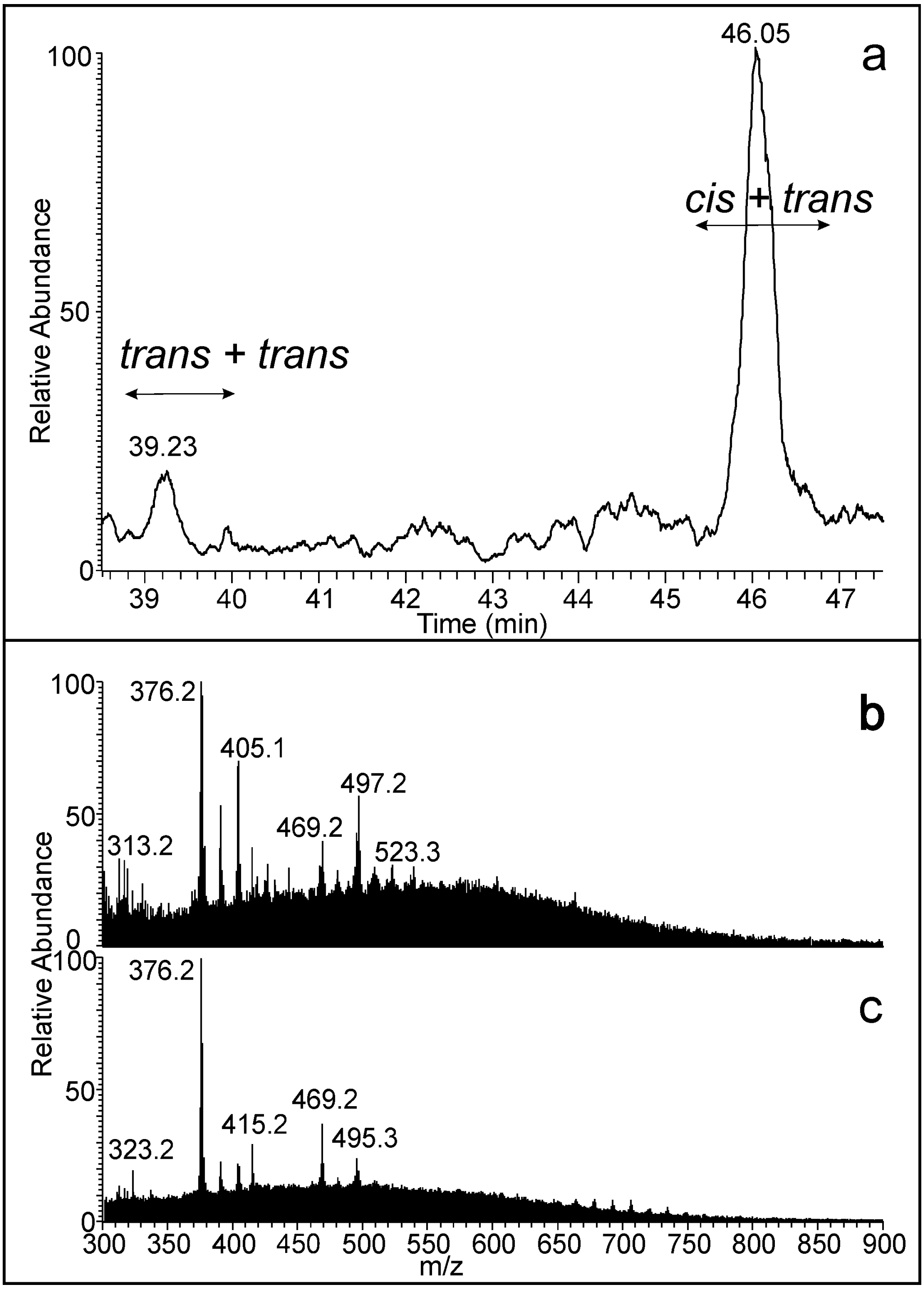

Figure S5. (a) Basepeak chromatogram of diunsaturated HCs from *P. americana*, (b) APCI-MS spectrum averaged within R_t 39.0-39.5 min range (two trans double bonds), and (c) APCI-MS spectrum averaged within R_t 45.8-46.4 min range (one cis double bond, one trans). The sample was separated in the solvent system III with a linear increase of toluene (B) in hexane (A) at 0.50 % B/min. The column temperature was 15°C.
